# Pathomechanism underlying the onset of scoliosis in a PNX broiler chicken model

**DOI:** 10.1186/1748-7161-8-S2-O3

**Published:** 2013-09-18

**Authors:** Yoichi Aota, Hayato Terayama, Tomoyuki Saito, Masahiro Itoh

**Affiliations:** 1 Department of Orthopaedic Surgery, Yokohama City University, Yokohama, Japan

## Purpose

The pinealectomy (PNX) in a chicken model consistently induces scoliosis with anatomic features that are similar to human adolescent idiopathic scoliosis (AIS). This experimental study attempted to improve the understanding of the mechanisms underlying the onset of scoliosis in a PNX broiler chicken model.

## Methods

A histomorphometric study was performed to analyze longitudinal bone growth and cancellous bone remodeling before the development of scoliosis. Static and dynamic parameters in cancellous bone and chondro-osseous junction of the 7^th^ thoracic vertebral body at nine days after hatching were compared between PNX chickens (n=9) and control chickens with no surgery (n=5).

## Results

The PNX resulted in a rapid and marked loss of cancellous bone volume (8 ± 1% versus 14 ± 2%, mean ± SD, p<0.005) and profoundly disrupted trabecular structure with increases in dynamic formative parameters, such as mineralizing surface, mineralization apposition rate and adjusted appositional rate. In the chondro-osseous junction, activated osteoclasts phagocitized degenerating chondrocytes, leaving a minimal amount of cartilage matrix and activated osteoblasts, thus losing their scaffolding for bone formation directly covering the hypertrophic zone cells. The osteoid surface and thickness in the chondro-osseous junction were significantly increased in PNX chickens (43 ± 14 % versus 12 ± 6 % and 4 ± 0.2µm versus 3 ± 0.4 µm). In the subjacent cartilage regions being protected from further resorption, abundant labeled cartilage remained with higher cellularity.

## Conclusions and discussion

It is known that fast-growing birds have a unique paradigm of rapid bone elongation with minimal metaphyseal bone production. A bone-forming surface exists at the front of cartilage ossification in the growth plate. Capillae of hypertrophic chondrocytes become included between the trabeculae of metaphyseal bone, and the overall thickness of the growth plate increases considerably in addition to distal expansion. Our results indicate that the unique mechanism for rapid bone elongation in chickens is more pronounced after PNX. PNX also induces high turnover osteoporosis, which may contribute to the development of scoliosis in the chicken.

## References

[B1] Machida M Cause of idiopathic scoliosis Spine 1999 24 2576 83 10.1097/00007632-199912150-00004 10635520

